# Congenital Thrombotic Thrombocytopenic Purpura: A Rare Cause of Recurrent Thrombocytopenia and Anemia

**DOI:** 10.7759/cureus.44988

**Published:** 2023-09-10

**Authors:** Mona Assiri, Asmaa AlMalki, Bayan AlHunif, Maha AlMofareh

**Affiliations:** 1 Pediatric Hematology/Oncology Department, Maternity and Children Hospital, Abha, SAU; 2 Pediatric Department, Maternity and Children Hospital, Abha, SAU; 3 Pediatric Hematology/Oncology Department, King Khalid University, Abha, SAU

**Keywords:** upshaw–schulman syndrome, adamts13, von-willebrand factor, thrombocytopenia, congenital thrombotic thrombocytopenic purpura

## Abstract

Congenital thrombotic thrombocytopenic purpura (cTTP) is a rare autosomal recessive microangiopathic disorder caused by inherited mutations in the ADAMTS13 gene. cTTP treatment involves infusing ADAMTS13-rich blood products like fresh frozen plasma (FFP) to replenish levels and prevent disease relapses. Alternative therapies like recombinant ADAMTS13, plasma-derived Factor VIII, or caplacizumab may be used for patients unable to tolerate FFP. We present a case of a five-month-old girl who had recurrent episodes of anemia and thrombocytopenia. She was diagnosed with cTTP based on the presence of low ADAMTS13 activity and the identification of a homozygous likely pathogenic variant in the ADAMTS13 gene. After receiving regular transfusions of FFP, our patient improved significantly and has been asymptomatic for 18 months with no transfusion complications.

## Introduction

Congenital thrombotic thrombocytopenic purpura (cTTP), also known as Upshaw-Schulman syndrome, is a rare microangiopathic disorder caused by inherited mutations in the ADAMTS13 gene [1]. In the absence of ADAMTS13, large von Willebrand factor (vWF) multimers accumulate, resulting in extensive platelet aggregation and multiorgan microvascular thrombi [2].

The mechanism of deficiency in immune TTP is caused by acquired autoantibodies against ADAMTS-13, whereas in fewer cases of cTTP, severe deficiency of ADAMTS-13 is recessively inherited [2]. cTTP represents less than 5% of all cases of TTP [1,2]. Clinical manifestations of cTTP are highly variable, ranging from mild nonspecific symptoms and isolated thrombocytopenia to severe neonatal hyperbilirubinemia with episodes of thrombocytopenia and microangiopathic hemolytic anemia (MAHA) [3,4].

We present a case of cTTP successfully treated with monthly FFP infusions.

## Case presentation

A five-month-old Saudi girl, born via spontaneous vaginal delivery following an uneventful pregnancy, was admitted to the neonatal intensive care unit due to anemia and jaundice caused by ABO incompatibility, which occurred shortly after birth. She was treated with exchange transfusion and discharged in a good state.

At the age of one month, she presented with a history of upper respiratory tract infection (URTI) and was found to have normocytic normochromic anemia and thrombocytopenia. Hematological investigations revealed a hemoglobin of 5 g/dl, reticulocytes of 2%, platelet count of 40/mm^3^, and a normal blood smear and coagulation profile. She was managed as disseminated intravascular coagulation (DIC) and received PRBC, platelet transfusions, and antibiotics. Two weeks later, Hb had improved to 11 g/dl, and platelets had increased to 160/mm^3^. The laboratory investigations are detailed in Table 1.

**Table 1:** Laboratory investigations of the patient ALT: Alanine transaminase; AST: Aspartate transaminase; LDH: Lactate dehydrogenase; ESR: Erythrocyte sedimentation rate; PT: Prothrombin time; APTT: Activated partial; thromboplastin time; INR: International normalized ratio; Hb: Hemoglobin

	Normal range	At the age of 1 month	At the age of 4 months	At the age of 5 months
Hemoglobin	1 month: 10.7-13.9 g/dl 2 months: 9.4-11.2 g/dl 5 months: 11.1-12.6 g/dl	5	8	5.88
Platelet	150-450×10^3^/mL	40	60	10.2
Creatinine	0.2-0.4 mg/dL	0.29		0.26
Total Bilirubin	<1.2 mg/dL	0.81		0.9
Direct Bilirubin	<0.2 mg/dL	0.15		0.12
ALT	13-45 U/L	23		27
AST	9-80 U/L	24		28
LDH	180-430 U/L	336		228
ESR	0-10 mm/hr	12		10
APTT	35-46.3 sec	36.6		38.2
PT	11.5-15.3 sec	17.6		16.5
INR	0.86-1.22 sec	1.05		1.1
Ferritin	50-200 ng/mL	250		198
Hemoglobin electrophoresis				
Hb A	96.8-97.8%	95%		
Hb F	<0.5%	1.8 %		
Hb A2	(2.2-3.2%)	2.7 %		

The third admission was at the age of four months due to pallor and ecchymosis. The Hb was 8 g/dl and the PLT was 60/mm^3^. She was admitted to our hospital at the age of five months with a complaint of epistaxis, described as a moderate amount of fresh blood stopped after compression. Her family denied any previous history of bleeding from other orifices, ecchymosis, easy bruising, gum bleeding, or oozing during IV-line insertion. They noticed that she had turned pale and that her activity level had significantly decreased due to irritability. There were no neurological or renal manifestations.

The patient had no prior surgery or a history of abnormal blood transfusion reactions. She was vaccinated in accordance with the Saudi vaccination schedule and had age-appropriate milestones. She was born to healthy consanguineous parents and has one sibling who is also healthy. There is no family history of a similar presentation or hematological diseases. The physical examination revealed that she was pale, not jaundiced, and had no dysmorphic features with normal growth parameters. She had a flat spot on the left side of the head (plagiocephaly). Vitally, she was stable. The remainder of the systematic examination was within normal limits.

**Figure 1:**
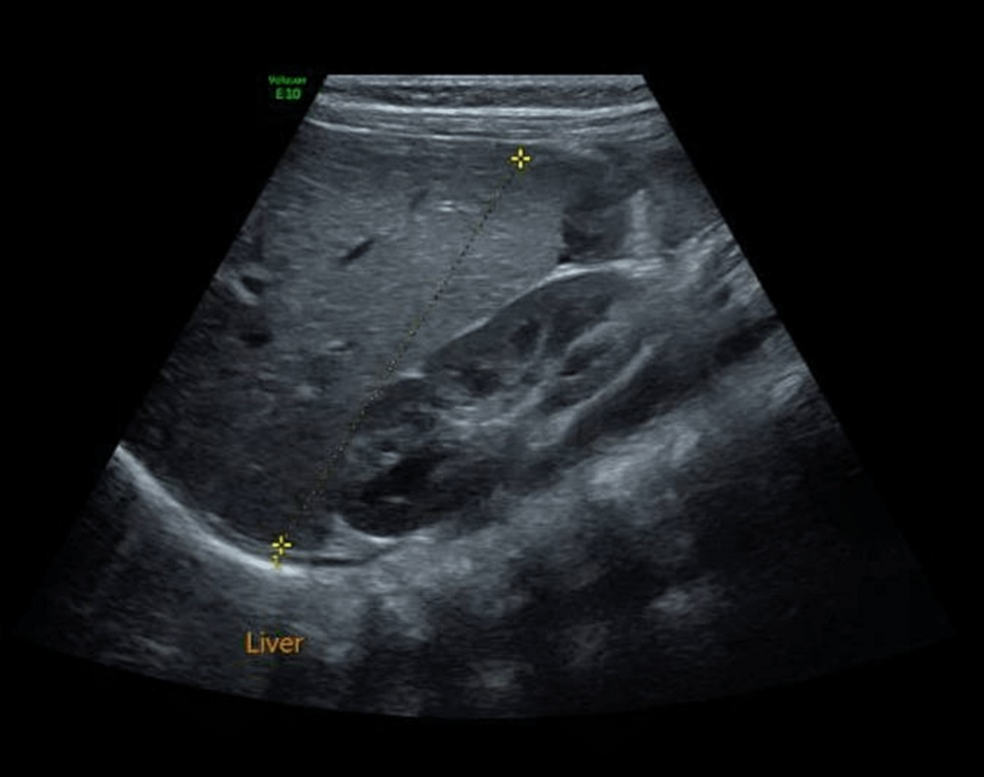
Normal ultrasound abdomen

Her CBC upon admission showed normocytic normochromic anemia (Hb of 5.88 g/dl) and thrombocytopenia (PLT 10.2/mm^3^). After receiving PRBC and platelet transfusions, Hb increased to 9.4 g/dl and PLT to 66/mm^3^. A few hours after the transfusion, the patient became pale and tachycardic, prompting an urgent CBC, which showed that Hb had decreased to 6.8 g/dl and PLT to 10.2/mm^3^. Urgent cranial and abdominal US were unremarkable (Figure 1).

**Figure 2:**
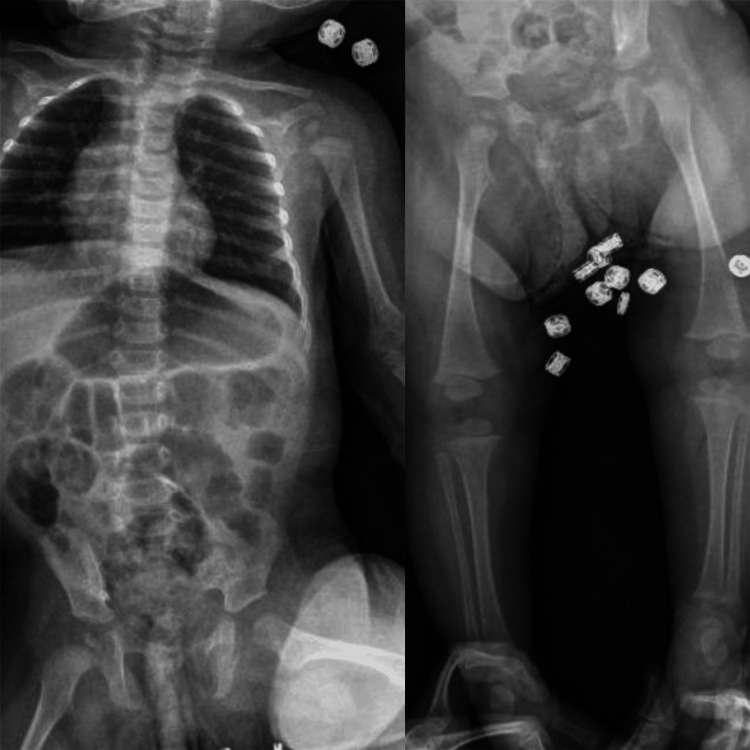
Normal skeletal survey The coin-like structures represent clothing buttons.

Further investigations were carried out as follows: peripheral blood smear was unremarkable, direct and indirect Coombs tests were negative, Hb electrophoresis was normal, and viral infections such as TORCH (Toxoplasma gondii, Rubella, Cytomegalovirus, and Herpes simplex virus), HIV (human immunodeficiency virus), and EBV (Epstein-Bar virus) were negative. The skeletal survey was normal (Figure 2).

**Figure 3:**
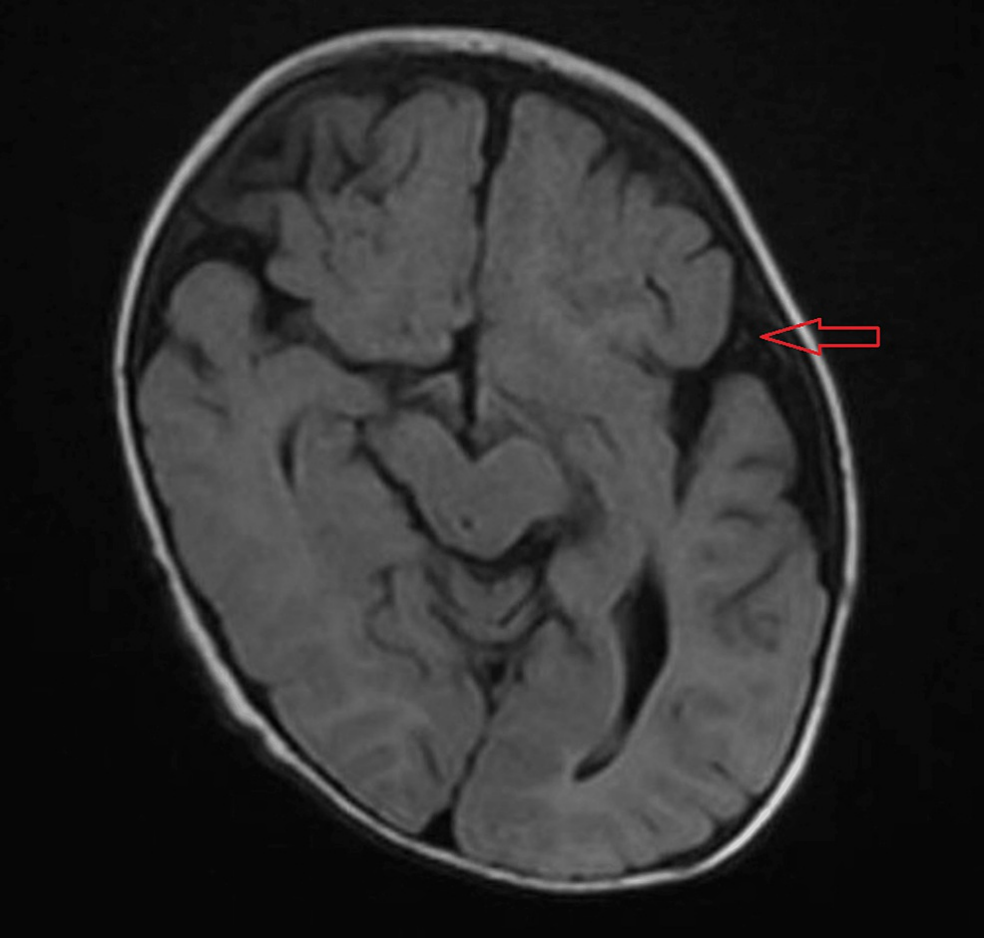
MRI brain without contrast Left hemicerebrum atrophy, as evidenced by the prominent ventricular system and extra-axial CSF spaces on the left side, most likely secondary to a brain insult (arrow)

Because of the abnormal head shape, an MRI brain was performed, which revealed left hemicerebrum atrophy, as evidenced by the prominent ventricular system and extra-axial CSF spaces on the left side, most likely secondary to brain insult. A small right parietal focal lesion with minimal positive blooming artifacts in susceptibility-weighted imaging (SWI) suggests a hemorrhagic nature, most likely a small cavernoma (Figure 3).

The bone marrow aspirate showed normocellular bone marrow with no abnormal cells. Finally, after ruling out malignancy and infectious causes, the level of ADAMTS-13 activity was measured and found to be 0.016 IU/ml (the normal range is 0.4-1.3 IU/ml), which strongly suggests TTP. Genetic analysis revealed the presence of a homozygous, likely pathogenic variant in the ADAMTS13 gene, confirming the diagnosis of cTTP. The ADAMTS13 variant c.1520G>A p.(Arg507Gln) causes an amino acid change from arginine to glutamine at position 507. She is currently receiving an FFP infusion of 10 ml/kg on a monthly basis and is doing well.

## Discussion

cTTP is an autosomal recessive thrombotic microangiopathy caused by a biallelic mutation in the ADAMTS13 gene, located on chromosome 9q34, resulting in severe ADAMTS13 deficiency (activity<10%) [5]. In the absence of anti-ADAMTS13 antibodies, the definitive diagnosis of cTTP is dependent on undetectable or severely deficient ADAMTS13 activity [6]. ADAMTS13 activity measurement should be considered in siblings of patients with documented cTTP [7]. More than 200 pathogenic ADAMTS13 variants have been identified [8]. The ADAMTS13 variant in our patient was c.1520G>A p.(Arg507Gln), which has previously been described as causing cTTP disease [8].

Childhood-onset cTTP affects 38% of cases diagnosed in a large cohort of 73 cases diagnosed in the United Kingdom (UK), with a median age of diagnosis of three years (range, 1 month to 12 years). Thrombocytopenia and MAHA were present in 100% and 29% of cases, respectively. Infection is the most common precipitant. Four neonatal patients had hyperbilirubinemia and thrombocytopenia [4].

The goal of cTTP treatment is to replenish ADAMTS13 levels. This can be accomplished through therapeutic plasma exchange, which is rarely required, or by infusing ADAMTS13-rich blood products such as FFP [9]. Treatment of the precipitant cause, such as infection, is crucial [4].

Severe neonatal jaundice and hemolysis may necessitate a blood transfusion [10]. In two case series from Japan and Norway, 42% and 45% of neonates, respectively, required exchange transfusions due to severe hemolysis and neonatal hyperbilirubinemia [11,12].

FFP infusion is the preferred treatment option for treating acute cTTP attacks and maintaining plasma levels of ADAMTS13 activity above 5%, which prevents disease relapses. According to the recent International Society of Thrombosis and Hemostasis (ISTH) TTP guidelines, FFP is typically administered at 10 to 15 mL/kg at three weekly intervals as maintenance therapy and daily for symptomatic patients until symptoms resolve and platelet counts normalize [9]. Patients who are unable or intolerable to plasma may be treated with alternative therapies such as recombinant ADAMTS13, plasma-derived Factor VIII, or caplacizumab.

Although recombinant ADAMTS13 (apadamtase alfa) is not commonly used in clinical practice, it can be used for patients who are unable to receive or benefit from plasma [13]. A randomized trial studying the use of recombinant ADAMTS13 is ongoing [14].

The ISTH TTP guidelines recommend against using FVIII concentrate for most patients with cTTP in remission due to a lack of clear evidence supporting its use, and the amount of ADAMTS13 in FVIII concentrates is quite low [15]. In contrast, a UK study of seven patients found plasma-derived factor VIII/VWF concentrates to be effective as a source of ADAMTS13 in patients who are intolerant or hypersensitive to FFP [16]. Factor VIII replacement product use on-demand or prophylactically was reported to be a successful treatment of cTTP in another US cohort [17].

Caplacizumab is a humanized monoclonal antibody that binds to VWF and inhibits the interaction of VWF multimers with platelet glycoprotein 1. It has been approved for the treatment of acquired TTP, particularly in patients with severe symptoms [18]. A single caplacizumab infusion was found to be effective in one patient with severe cTTP [19].

A previous case report from Saudi Arabia described a two-year-old girl with a cTTP phenotype caused by a novel homozygous ADAMTS13 mutation (c.2882delC; p.Cys962Alafs*3). She was successfully treated with FFP infusion after presenting with neonatal hyperbilirubinemia, recurrent episodes of thrombocytopenia, and MAHA [20].

## Conclusions

Our goal in publishing such a rare case is to make clinicians aware of this potential presentation in order to prevent delays in diagnosis and treatment. cTTP diagnosis should be considered in the presence of recurrent episodes of thrombocytopenia in neonates after ruling out neonatal hemolysis and other thrombotic microangiopathies. The prophylactic transfusion of FFP has been linked to significant improvements in symptoms and outcomes.
